# Healthcare Cost and Outcomes Associated With Surgical Site Infection and Patient Outcomes in Low- and Middle-Income Countries

**DOI:** 10.7759/cureus.42493

**Published:** 2023-07-26

**Authors:** Fernando Costabella, Keval B Patel, Adedimeji V Adepoju, Purnima Singh, Hussein Attia Hussein Mahmoud, Awais Zafar, Tirath Patel, Ninad A Watekar, Navya Mallesh, Moiz Fawad, Dily T Sathyarajan, Kiran Abbas

**Affiliations:** 1 Department of Pediatrics, Hospital General Dr. Manuel Gea Gonzalez, Mexico City, MEX; 2 Department of Surgery, Narendra Modi Medical College, Ahmedabad, IND; 3 Department of Surgery, St. George's University, West Indies, GRD; 4 College of Medicine, Gulf Medical University, Ajman, ARE; 5 Department of Diagnostic Radiology, Heliopolis Hospital, Cairo, EGY; 6 Department of Psychiatry, Sahiwal Medical College, Sahiwal, PAK; 7 Department of Surgery, American University of Antigua, St. John, ATG; 8 Department of Surgery, Davao Medical School Foundation, Davao, PHL; 9 Department of Surgery, St. Martinus University, Willemstad, CUW; 10 Department of Neurological Surgery, King Saud Hospital, Unaizah, SAU; 11 Department of Pediatric Surgery, Indira Gandhi Institute of Child Health, Bengaluru, IND; 12 Department of Surgery, Government Medical College, Thrissur, IND; 13 Department of Community Health Sciences, Aga Khan University, Karachi, PAK

**Keywords:** scarce resource allocation, financial impact, healthcare costs, economic burden of healthcare, surgical site infections (ssi)

## Abstract

Surgical site infection (SSI) is a growing global concern. The principal explanation for this is its adverse clinical outcomes, such as morbidity and mortality. However, the link between the economic burden of SSIs and patient outcomes needs to be sufficiently characterized. This review aims to describe the financial implications of SSIs on patient outcomes in low- and middle-income countries (LMIC). Despite the heterogeneity in study designs from multiple LMIC countries, there is a significant correlation between SSI-associated healthcare costs from increased length of stay (LOS), readmissions, reoperations, and adverse patient outcomes. This varies based on the size, degree of infection, or other patient comorbidities. SSIs are much more prevalent in LMICs. The additional financial burden incurred in managing SSIs reinforces the need to prioritize practicing interventions to prevent this complication, which resource-limited health institutions are unequipped to do and consequently have significant adverse patient outcomes.

## Introduction and background

The term "surgical site infection" (SSI) was first inaugurated in 1992. According to the Centers for Disease Control and Prevention, it refers to a wound infection that develops within 30 days after an operative procedure or from an implant left in place for more than a year [1], disrupting the surgical incision itself or any of its surrounding anatomy [2]. SSI can be further categorized based on the depth of tissue involvement [3].

SSI is the most known complication of postoperative procedures, and it causes mental and physical suffering in patients with delayed recovery [4]. SSI is linked to multiple devastating outcomes that lead to higher healthcare-associated costs due to extended hospital stays and additional surgical expenses. Post-discharge emergency room visits have more than doubled because of SSI [5]. The complexity of the disease hinders medical and surgical management, thus leading to significant consequences in increased morbidity and mortality [6-9].

Despite the paucity of data available from LMICs, physicians still agree that hospital-acquired infections are due to a lack of appropriate and safe patient-centered care [8]. This impression is supported by high transportation costs or remote hospital destinations, leading to arduous follow-ups in LMICs [9]. Furthermore, nonmodifiable factors such as age, diabetes-mellitus, and immunosuppression, as well as modifiable factors that include smoking and obesity, notably contribute to the causative risk of SSI [3]. Antimicrobial resistance is by far the most bothersome etiology that impedes progress in the medical field [7], especially in surgeries.

Acquiring SSI in LMICs led to an exponential financial catastrophe, where healthcare expenditure on such events would exceed 10% compared to an annual household [10]. The economic consequences depend on the type of causative agent, each having its unique pathogenesis and clinical outcomes [11]. To mitigate the financial burden LMICs suffer, initiatives have been made to encourage investments in technologies for low-resourced health systems [12]. High-income countries (HICs) have shown a decrease in reported patients with SSIs after following the national SSI surveillance (nSSIS) programs [13]. It is noted that poor compliance with the current guidelines in LMICs hinders successful outcomes [14]. nSSIS programs will improve patients’ well-being if implemented in LMICs by promoting leadership to encourage safe surgical procedures [13]. In this review, we aimed to explore the healthcare-associated costs of SSI incidences and their impact on patient outcomes.

## Review

Healthcare cost of SSIs

Despite decades of extensive focus and progress in medical treatments, SSIs remain a significant concern for patients and health institutions [15-17]. According to a study conducted in 2017, the reported occurrence of SSIs has remained largely unchanged over the past five decades, implying its significance and the need to explore the economic burden of SSIs on healthcare and individual patients [15].

SSIs are also major causes of morbidity, prolonged hospital stays, extended antibiotic therapy, unplanned readmissions, poorer long-term patient outcomes, and additional surgeries [16-18]. These burden the healthcare system and strain its efficiency. The growing pressure to manage healthcare resources and address rising costs has reduced reimbursements for treating preventable complications such as SSIs [19]. In Ghana, the availability of ambulances is limited, meaning the transport cost falls on the patient’s family. This also results in lost time from earning wages that are often needed to support other family members [20].

There has been growing attention to reducing healthcare costs by preventing SSIs. SSI-associated healthcare expenses (direct or indirect) have roughly doubled [16]. Per a cohort study that compared pediatric patients with and without preventable SSIs, costs were 36% higher for the group with SSIs, and the duration of stay was up by approximately 11 days [17]. Moreover, a study from India that focused on the economic burden of SSIs among patients post-C-section revealed that the total morbidity cost tripled, and hospital stays were 10 days longer than the average [18].

A systematic review in 2020 explored the impact of SSIs in LMICs, with SSIs found to be responsible for 38% of deaths. Compared to HICs in Europe, LMICs experienced a higher incidence of SSIs, with the majority of the costs falling on the patient, where healthcare expenses related to this event exceed 10% of the household’s annual expenditure [9]. Some contributory factors leading to high costs associated with SSI are inadequate surgical infrastructure, human resources, and equipment [21]. A study on hospitals in Uganda revealed that the surgical burden exceeded the hospitals’ workforce and infrastructure [22]. Overcrowded wards were common, with patients having to share beds, sleep on the floor, or even outside the ward. Delays in providing the necessary surgical intervention were primarily caused by high patient volumes, limited healthcare personnel, and the unavailability of medical equipment and drugs [22].

Numerous barriers to SSI prevention were identified by a study done in a tertiary care hospital in Ethiopia [23]. These included limited antiseptic materials and water supplies, a lack of appropriate guidelines and training for SSI management, unsatisfactory SSI tracking, and less infection prevention control communication with surgical staff. Compliance in terms of preoperative cleansing (26.3%), methicillin-resistant *Staphylococcus aureus* screening (0%), and temperature (57.9%, 47.3%) monitoring was found to be poor and discouraging [23]. Another study in northeast Ethiopia found that many healthcare professionals exhibited inadequate knowledge and engaged in unsafe practices concerning infection prevention [24].

Barriers to lowering the SSI rate in LMICs include poor surveillance of infection incidence and a lack of guidelines for prophylactic antibiotics, resources, trained staff, and basic hygiene [25,26].

Burden of SSIs

In the last two decades, the statistics of SSIs have diverged between LMICs and industrialized nations due to the development of new surgical methods, improved training, and changes in socioeconomic indices. There are fewer hospital infection control committees with financial backing in LMICs compared to higher-income nations that have organizations and resources to implement specific initiatives. According to data from India, Brazil, and Mexico, the pediatric SSI rates were significant, at 5.4%, 6.7%, and 18.7%, respectively [27]. The duration of surgery, the level of wound contamination, a shortage in hospital infection management as well as antibiotic regulations, the more significant expense in therapy, a delay in detection and treatment, the lack of competent staff and supplies, and budgetary limitations are merely a few in the multiple variables that contribute to a high rate in SSIs on pediatric patients [28,29].

Bakshi et al. reviewed data gathered over three decades on the outcomes of infant cardiovascular surgery for congenital heart conditions. They detected that lengthier hospitalization, postponed thoracic closure, after-surgery antibiotic use, wound infection development, and reintubation are risk factors for developing SSIs. Establishing and executing infection-control protocols is an effective method for preventing SSIs [30]. More recently, a prospective observational analysis was applied to review a specific pediatric surgical care program. The development of a detailed worldwide registry on important outcome metrics and nursing instruction aimed at recommended procedures were among this program's core elements. The single most evident outcome of these efforts was a significant drop in SSIs from 11.1% in 2010 to 2.4% in 2013 [31]. De Jonge et al. developed a novel SSI program for people to preserve their health after elective abdominal surgery in adults [32].

Patient outcomes associated with SSIs

Length of hospital stay (LOS), postoperative mortality, and additional procedures needed, including re-operations, are patient outcomes directly related to healthcare expenditure [33]. The economies of these developing nations are put under a more significant financial strain due to all of the following factors (Table 1) [9,33-40].

**Table 1:** Health outcomes associated with SSIs LOS: length of hospital stay, SSIs: surgical site infections, QoL: quality of life

Health Outcome	Key Findings	References
LOS	Increased LOS is consistently associated with SSIs. Extra days in the hospital varied from one additional day for limb amputations to 16 additional days for rectal surgery.	Fenny et al. 2020 [33]
Productivity loss	Patients with SSIs reported a higher number of missed days and greater indirect costs compared to patients without SSIs.	Fenny et al. 2020 [33]
Reoperations	SSIs and incision sites were significantly associated with the need for re-operation. Other predictors included postoperative antibiotic prescription, emergency surgery, duration of operation, and comorbidities.	Misha et al., 2021 [34]
Postoperative mortality	SSIs contributed to 38% of postoperative deaths. Postoperative mortality is the third highest contributor to global deaths, with SSIs playing a significant role.	Nepogodiev at al., 2019; Astagneau et al., 2001; Gheorghe et al., 2015; Badia at al., 2017 [35-38]
Patient QoL	SSIs have a substantial negative impact on Quality of Life (QoL), affecting physical, psycho-social, and financial well-being, as well as doctor-patient relationships.	Avsar et al., 2021 [40]

LOS

The estimated hospital expenditure was derived from LOS and the mean departmental cost per 24 hours. The mean LOS was 4.6 in the control group compared to 9.2 in patients with SSI (p=0.003). The finding mentioned above of increased LOS was consistently found to be present across different types of surgical procedures. The total number of additional days in the hospital varied from a mean of one extra day for limb amputation to a mean of 16 additional days for rectal surgery [33].

Productivity Loss

Productivity loss refers to the opportunity expenses incurred by patients and their carers due to hospitalization, such as lost income owing to illness or caring tasks during admission and the ensuing 30 days after discharge. For practically all surgical procedures, individuals with SSI reported a higher number of days missed and higher indirect expenses (95% CI = $381-$893) compared to the non-SSI patients (95% CI = $185-$730) [33].

Re-operations

A prospective cohort study in Southwest Ethiopia studied the association with reoperation among patients with and without SSIs. Half of the 251 participants included in the study were females (126). Many patients in the study developed SSIs, 53 (21.1%). The study showed that 29 patients (11.6%) returned to the operating rooms. The Cox regression study demonstrated that SSI (AHR (95% CI) = 7 (3.16-15.72)) and incision site [AHR (95% CI) = 2.5 (1.14-5.41)] are significantly related to the need to re-operate. It should be noted that the previously indicated risk is affected independently by predictors such as postoperative antibiotic prescription, emergency surgery, duration of operation, and comorbidities [34].

Postoperative Mortality

According to a study, at least 4.2 million individuals globally die within 30 days of surgery each year, with LMICs accounting for half of these deaths. This makes postoperative mortality the third highest contributor to global deaths. Annually, the number of deaths within 30 days postoperatively surpasses the combined death toll of HIV, malaria, and tuberculosis [35]. Among these patients, SSI contributes to 38% of postoperative deaths [36]. SSIs, due to their effect on patient morbidity and mortality [37], are recognized globally as notable healthcare problems with substantial financial consequences [38,39].

Patient QoL

Despite having substantial adverse effects on patient outcomes, little or no research is available on the impact of SSI on patient QoL, especially in LMICs. In this section, we mentioned the results of a study that revealed the impact of SSIs on QoL. Both qualitative and quantitative measures were analyzed, mainly using SF-36 questionnaires. According to the findings of the few studies, SSI has a considerable negative influence on patients' QoL and impacts their physical, psycho-social, and financial well-being, as well as the doctor-patient connection [40-47]. A nested case-control study at the University of Iowa Hospitals and Clinics revealed that factors like LOS before surgery and postoperative cerebrospinal fluid leak increase the risk of SSIs. SSIs lead to extended hospitalization, readmissions, reoperations, and higher mortality rates, emphasizing the importance of preventing them for better patient outcomes and reduced healthcare burdens [45-49].

Although there are international recommendations for SSI prevention, it can be challenging to implement them in various clinical settings, especially in LMICs because of a lack of resources and an unorganized infrastructure [42-52].

Financial consequences experienced by patients who develop SSIs

A recent retrospective study conducted on pediatric patients revealed that the average cost of SSIs amounted to $136,950. In 50% of the cases, reentry procedures were required after the detection and treatment of SSIs, resulting in an additional median cost of $116,342. The study identified a clear correlation between elevated intervention and divisional hospital expenses and the occurrence of SSIs following cardiac surgery [41].

LMICs and SSIs

Although there are international recommendations for SSI prevention, it can be challenging to implement them in various clinical settings, especially in LMICs, because of a lack of resources and an unorganized infrastructure [42]. This section highlights the differences in patient outcomes between high-, medium-, and low-income nations (Table 2).

**Table 2:** SSIs in LMICs as compared to HICs HDI: human development index, SSI: surgical site infections, LMICs: low- and middle-income countries, HICs: high-income countries

Study Focus	Key Findings	References
SSI in relation to HDI	Countries with a lower HDI have a higher incidence of SSIs, particularly for "dirty" surgeries. Patients in these countries are at the greatest risk of developing SSIs, and a significant number have antibiotic-resistant infections.	GlobalSurg Collaborative, 2018) [43]
SSI rates in Europe	SSI rates in Europe varied widely, from 1.5% to 20%, likely due to inconsistencies in data collection methods and surveillance criteria. The economic costs were substantial, ranging from 1.47 to 19.1 billion Euros. The study suggested that the true rate and economic burden of SSIs have been underreported.	Leaper et al., 2004 [44]
Comparison of SSI costs in LMICs and HICs in Europe	The additional cost of SSIs varied widely in both settings, with LMICs ranging from $174 to $29,610 and high-income European countries ranging from $21 to $34,000. This study emphasizes the substantial cost burden imposed by SSIs in LMICs.	Monahan et al., 2020 [9]

For instance, an international prospective study revealed that countries with a lower human development index (HDI) have a higher incidence of SSIs compared to countries with a higher HDI. Low-HDI countries had the highest SSI rates, particularly for "dirty" surgeries. Patients in low-HDI countries were also at the greatest risk of developing SSIs. Additionally, a significant number of patients in all HDI groups had antibiotic-resistant infections. The study emphasizes the need for urgent research in LMICs to reduce SSIs and address antibiotic resistance [43]. David et al. undertook a retrospective review of reported SSI rates in Europe to estimate the scale of the problem and its economic burden. The estimated SSI rates varied widely (1.5-20%) due to inconsistencies in data collection methods and surveillance criteria. The economic costs of SSIs were found to be substantial, ranging from 1.47 to 19.1 billion Eurodollars. The study highlights that both the true SSI rate and its economic burden have been underreported in the literature. The authors recommended improvements in study design, data collection, analysis, and reporting to accurately assess SSI rates and evaluate cost-effective measures in the future [44].

Monahan et al. undertook a systematic review to critically appraise studies on the cost of SSIs in both LMICs and HICs in Europe [9]. They identified and reviewed studies from 15 LMICs and 16 European countries. The additional cost of SSI varied widely in both settings, with LMICs ranging from $174 to $29,610 and European countries ranging from $21 to $34,000. The study emphasized that SSIs impose a substantial cost burden on both LMICs and HICs in Europe [9].

Increased SSI incidence raises total medical expenses, including direct and indirect healthcare costs resulting from post-discharge medical treatment. Because of a lack of infrastructure and norms for safe practices, low-income countries have higher overall expenses than high- and middle-income countries.

Mitigating SSIs

Strategies for reducing SSIs and enhancing patient outcomes are multi-faceted. They involve maintaining the flow of traffic within the operating room to minimize contamination and diligent care of surgical wound dressings to protect against external contaminants and promote healing [52-54]. It's also important to regulate blood glucose levels in the immediate postoperative period and discontinue antibiotics within 24 hours after surgery to prevent the rise of antibiotic-resistant bacteria [52,53]. Education is crucial; regular training for surgeons and perioperative staff can help lower the risk of SSIs. This goes hand-in-hand with implementing evidence-based practices and multifaceted strategies to reinforce adherence to interventions that reduce SSIs [52].

Preventing surgical infections effectively also requires systems redesign to lessen risk factors and optimize evidence-based care processes. Core measures, such as the judicious selection, timing, and duration of antimicrobial prophylaxis, glucose control in cardiac surgery, and appropriate hair removal techniques, are essential. In cases of heavily contaminated wounds, delaying primary closure can help minimize the risk of SSIs. Furthermore, patient engagement, such as encouraging hand hygiene practices, can enhance compliance and reduce healthcare-associated infections [52].

## Conclusions

SSIs increase morbidity, mortality, and long-term disability in patients undergoing surgeries. These adverse outcomes are exponentially heightened in LMICs. This is because even though many SSIs are preventable, resource-limited institutions in LMICs face daunting challenges in implementing SSI-reducing strategies and managing active SSIs, and the economic burden inadvertently falls directly on patients who are primarily poverty-stricken or underprivileged.

The clinical deterioration associated with SSIs and the added unforeseen costs of management is strongly implicated with negative patient outcomes. There is a lack of universally accepted specific directives for preventing and managing SSIs specifically created for resource-limited health institutions in LMICs. Further studies should develop evidence-based strategies and standardized operative plans for these institutions. Additionally, given the nonuniformity in research design, a single multicenter study focused on LMICs will allow accurate data collection and generalizability of results. Lastly, our findings indicate a critical need for a clear and concise guideline for the perioperative prevention and postoperative management of SSIs, which will significantly reduce the incidence of this preventable surgical complication in LMICs.
